# Electrical Characterization of Microelectromechanical Silicon Carbide Resonators

**DOI:** 10.3390/s8095759

**Published:** 2008-09-17

**Authors:** Wen-Teng Chang, Christian Zorman

**Affiliations:** 1 Department of Electrical Engineering, National University of Kaohsiung, No. 700, Kaohsiung University Road, Nan-Tzu District, Kaohsiung 811, Taiwan; E-mail: wtchang@nuk.edu.tw; 2 Department of Electrical Engineering and Computer Science, Case Western Reserve University, 10900 Euclid Avenue, Cleveland, OH 44106, USA; E-mail: caz@case.edu.tw

**Keywords:** MEMS resonator, Silicon carbide, Gas rarefaction, Duffing effect, Temperature coefficient

## Abstract

This manuscript describes the findings of a study to investigate the performance of SiC MEMS resonators with respect to resonant frequency and quality factor under a variety of testing conditions, including various ambient pressures, AC drive voltages, bias potentials and temperatures. The sample set included both single-crystal and polycrystalline 3C-SiC lateral resonators. The experimental results show that operation at reduced pressures increases the resonant frequency as damping due to the gas-rarefaction effect becomes significant. Both DC bias and AC drive voltages result in nonlinearities, but the AC drive voltage is more sensitive to noise. The AC voltage has a voltage coefficient of 1∼4ppm/V at a DC bias of 40V. The coefficient of DC bias is about -11ppm/V to - 21ppm/V for poly-SiC, which is more than a factor of two better than a similarly designed polysilicon resonator (-54 ppm/V). The effective stiffness of the resonator decreases (softens) as the bias potential is increased, but increases (hardens) as drive voltage increase when scan is from low to high frequency. The resonant frequency decreases slightly with increasing temperature, exhibiting a temperature coefficient of -22 ppm/°C, between 22°C and 60°C. The thermal expansion mismatch between the SiC device and the Si substrate could be a reason that thermal coefficient for these SiC resonators is about twofold higher than similar polysilicon resonators. However, the Qs appear to exhibit no temperature dependence in this range.

## 1. Introduction

MEMS-based resonators have attracted the attention of the integrated circuits (IC) industry because of their high quality factors (Qs) and their capacity for integration with silicon-based integrated circuits [1, 2]. Silicon-based MEMS resonators are used as timing references in applications that require a device technology that can range from very low to ultra-high frequencies. Silicon carbide (SiC) is a promising material for RF MEMS because it has a high Young's modulus-to-density ratio, resulting in an acoustic velocity that is significantly above that of Si [3, 4]. Furthermore, SiC is more resistant to mechanical wear, more electrically stable at higher temperatures, and significantly more inert to environmental conditions than Si, making it a potential substitute for Si in harsh environment applications [5]. Among more than 100 known polytypes, cubic 3C-SiC is the only polytype that can be grown on single crystal Si substrates, enhancing the possibility of integrating micromachined SiC resonators with silicon microelectronics.

Many of the essential characteristics of Si-based MEMS resonators, such as operating frequency and Q, are sensitive to environmental conditions, such as gas damping, ambient humidity and temperature. Gas damping causes a shift in the resonant frequency because it changes the overall frequency response of the oscillation system [6]. For Si-based devices, exposure to air can cause a drift in resonant frequency over time due to surface oxidation [7]. Fortunately, gas damping can be made negligible when the operating pressure is reduced to beneath a critical point pressure [8–11]. Packaging under an inert atmospheric or vacuum can improve or eliminate environmentally-induced performance variations. However, these solutions cannot prevent temperature-related instabilities due to heat transferred to the resonator from on-chip circuitry. In contrast to quartz crystal resonators that consist of two-port piezoelectric devices commonly used in electronic circuits, Si-based MEMS resonators usually operate using an electrostatic method to actuate the shuttle and sense its motional current. To extract such small signals, Si-based MEMS resonators require an extra port to bias the device and amplify the motional signal [1]. However, extreme biasing can cause nonlinearities at large amplitudes, and cause the nominal Q and resonant frequency to shift. In view of these effects in Si-based devices, this work aims to characterize the performance of 3C-SiC MEMS lateral resonators in terms of resonant frequency and Q as they relate to pressure, temperature and applied voltages.

A number of techniques have been used to actuate and sense the motion of microscale resonators for the purpose of evaluating the Q and resonant frequency. Optical detection techniques using laser-based photodetectors to detect the oscillation amplitude have been used to acquire the resonant frequency and Q of MEMS-based resonators [12–14]. Others have determined the Q by analyzing the decay of a vibrating cantilever driven by electrostatic force [15]. A common approach to study Si resonators uses electronic amplifiers in conjunction with electrostatic actuation [16]. The research presented in this paper follows such an approach because it enables the evaluation of SiC resonator performance under a range of environmental testing conditions.

Unlike Si-based devices, much less has been published on the topic of SiC resonator performance. Several papers have reported the Q values and resonant frequencies of polycrystalline 3C-SiC (poly-SiC) lateral resonators, but the effects of bias voltage and ambient temperature on performance were not presented [17, 18]. The measurement of Q and resonant frequency as a function of temperature has been reported for poly-SiC lateral resonators, but the characterization technique utilized SEM imaging, therefore the reported values of Q are, at best, upper bound estimates because frequency spectra were not obtained [19]. The Q of clamped-clamped poly-SiC microbridges has been reported; however, the Q values for these devices was so low (128) that many important operational parameters were not reported [20]. The research reported in this paper seeks to increase the understanding of SiC lateral resonators by characterizing the performance of these devices under a variety of environmental conditions using an electronics-based characterization technique capable of accurately determining resonant frequency and Q.

## 2. Experimental Setup

### 2.1 Device Fabrication and Measurement Technique

**Figure 1.**
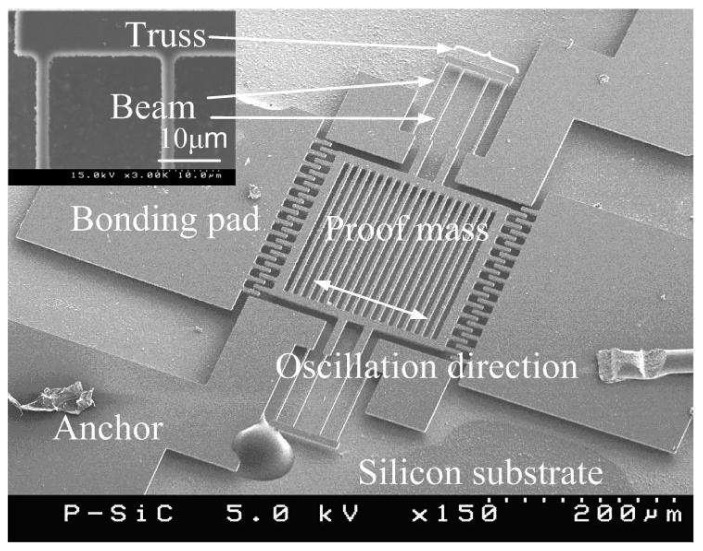
Plan-view SEM micrograph of a polycrystalline 3C-SiC, flexural mode lateral resonator *Device D_f_1*.

**Figure 2.**
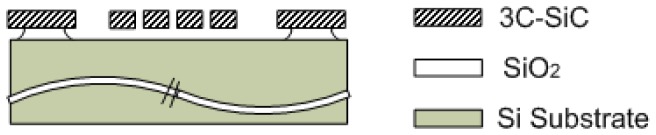
Schematic cross-section of the 3C-SiC lateral resonators used in this study.

The single-crystal (110) and polycrystalline (111) SiC films in this study were deposited by atmospheric pressure chemical vapor deposition (APCVD) and low pressure chemical vapor deposition (LPCVD), respectively. The single-crystal 3C-SiC, folded-beam, lateral resonators, denoted *Type K*, were fabricated from SiC-on-Insulator substrates in which the buried SiO_2_ film was used for the sacrificial layer and substrate electrical isolation. The polycrystalline 3C-SiC, folded-beam lateral resonators were made from undoped LPCVD films deposited on SiO_2_ sacrificial layers and are denoted as *Type D_f_* in this work. Figure 1 is a SEM micrograph of *Device D_f_1* and Figure 2 is a schematic cross-section of the 3C-SiC lateral resonator used in this study. The micrograph in Figure 1 was taken after experimentation was complete and the resonator chip was debonded from the test circuitry. The fabrication processes for these devices have been detailed elsewhere [11].

**Figure 3.**
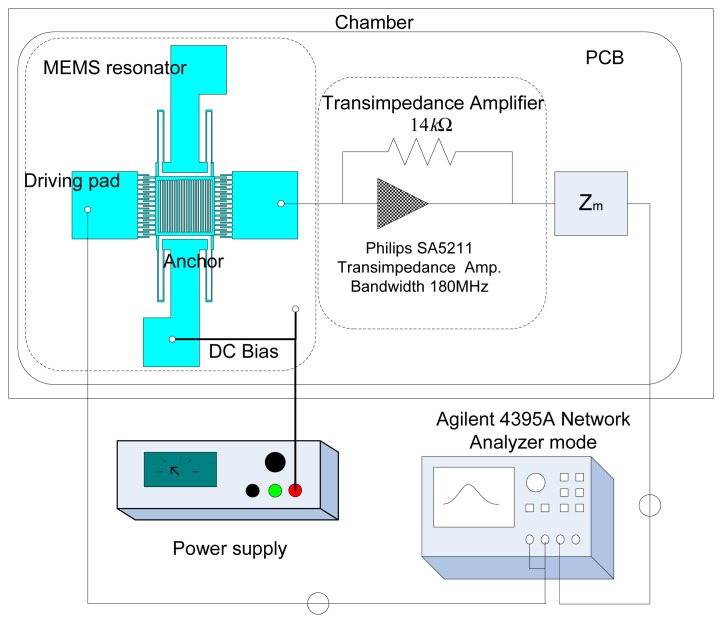
Schematic diagram of the electrical measurement setup.

The principal technique used in this work to acquire resonant frequency and Q is based on a custom-built, PCB-based transimpedance amplifier circuit. A schematic diagram of the setup is shown in Figure 3. The resonators were electrostatically actuated and the resulting motional current was detected and amplified by a transimpedance amplifier positioned next to the MEMS chip. When in oscillation, the MEMS resonator generates a motional current that is converted to a voltage signal by the transimpedance amplifier. The Philips SA5211 transimpedance amplifier was chosen because it uses a BJT differential amplifier that is capable of providing a low noise output signal, and the bandwidth of this amplifier reaches 180 MHz which meets the desired measurement range. The circuit was used to measure the total Q. The transimpedance amplifier approach enables frequency spectra to be acquired electronically using conventional actuation and measurement techniques.

**Figure 4.**
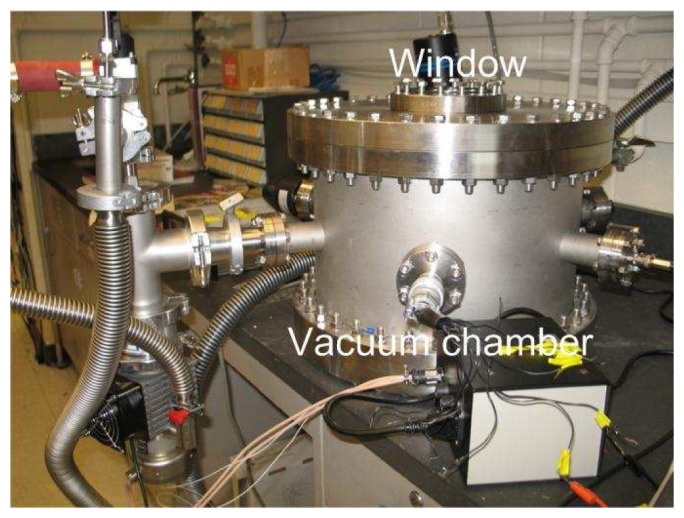
Photograph of the custom-built vacuum testing chamber. The chamber is equipped with a transparent window to enable radiative heating from outside the chamber, thus reducing electrical noise.

Ambient damping was characterized by operating the devices in a customized vacuum chamber capable of operating at pressures both above and below the critical point. The gain of the PCB-based testing circuit is measured by an Agilent 4395A network/spectrum analyzer. The network analyzer mode provides both magnitude and phase plots that help identify the resonant frequencies of the MEMS device. Figure 4 shows a photograph of the custom-built vacuum testing chamber used in this study. The chamber is equipped with a transparent window to enable observation of the resonator using a long working distance microscope as well as radiative heating of the device through the window using an external lamp. The chamber is outfitted with five ports for various external connections. Two of the ports are for electrical connectors: one for low noise connectors to the network analyzer, the other for power connections via multiple electrical wires. A third port is a high current feedthrough. The other two ports are used for the pressure gauge and the fore-line of the pumping system. The pressure gauges include a capacitance manometer and an ion gauge for low and high vacuum measurements, respectively. The vacuum system consists of a mechanical pump and a diffusion pump. The base pressure of the system with the PCB-based electronic testing circuitry mounted inside is roughly 30 μTorr when both pumps are operating and unthrottled.

### 2.2 Setup for Temperature Testing

**Figure 5.**
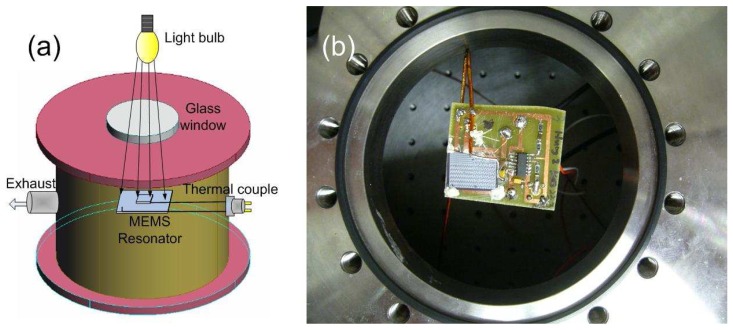
The temperature testing setup: (a) Schematic of the heating source and (b) optical photograph showing the position of the PCB that contains the test chip and associated electronics with respect to the chamber window.

An incandescent light bulb positioned outside the vacuum chamber and above the vacuum chamber window as shown in Figure 5(a) was used to heat the sample. Radiative heating was selected as the heating method because the radiative heat source could be positioned outside the vacuum chamber thus eliminating a source of electrical noise that could affect the measurement results. Lamp-based heating also allows for selective shadowing of the testing circuitry thereby heating only the resonator chip. Since radiative heating of the sample was employed, the maximum achievable temperature was limited by the radiation efficiency of the light bulb and the optical absorption of the substrate. The maximum practical testing temperature was limited by the maximum stable operating temperature of the transimpedance amplifier, which was 85°C. A direct temperature measurement of an individual resonator itself is technically challenging, so in this work temperature was measured at a conveniently accessible nearby location on the chip. Each chip contained approximately 90 individual resonators. Figure 5(b) is a photograph taken from the top of the chamber showing the technique used to detect temperature of the chip. A thermocouple makes contact with the surface of the chip and is linked to a thermometer outside the chamber through a multi-port feedthrough connector.

## 3. Result and Discussions

### 3.1 Gas rarefaction effect

**Figure 6.**
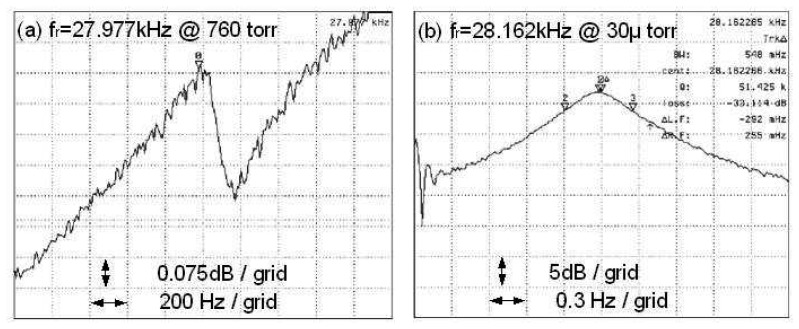
Magnitude plots for a device tested at: (a) atmospheric pressure and (b) 30 μTorr. Note that the amplitude scale in (a) is smaller than (b) and larger than (b) for the frequency scale because the Q in (a) is much smaller at atmospheric pressure.

Figures 6(a) and (b) are magnitude plots for a single crystalline 3C-SiC device *Device K1* that were measured at atmospheric pressure and at 30 μTorr, respectively. Table 1 illustrates the relationship between measured resonant frequency and ambient pressure for this device. These data show that the resonant frequency drops by 185Hz as the operating pressure is increased from 30 μTorr to 760 Torr. Note that the frequency scale in Figure 6(a) is 200 Hz/grid and the amplitude scale is 0.075 dB/grid because the Q is small at atmospheric pressure.

It is well known that for lateral resonators operated at pressures in the milliTorr to Torr range, ambient damping comprises a significant portion of the total dissipated power (i.e., 1/Q). A previous report showed that this loss is negligible for Si-based devices when the pressure is reduced below a critical point [11]. This effect can be understood by examining the velocity profiles at the surface of the resonator. At low pressure, there exists a velocity profile on the surface of a laterally moving MEMS resonator [6]. The damped spring-based oscillation system is described by:

(1) $$mx^{\prime \prime }+cx^{\prime }+kx=f(t)$$

where *m* is the effective mass of resonator, *c* is the damping coefficient that accounts for both ambient damping and internal damping within the structural material, *k* is stiffness coefficient and *x* is displacement. The resonators used in this study are driven to oscillate laterally in the plane of their substrates. For a normal viscous damping, the gas velocity is zero at the gas-surface interface (no-slip condition) but at low pressure or very narrow gaps, the gas rarefaction effect must be considered and the velocity is non-zero (known as the slip condition) at the interface. A compact damping can be described by Navier-Stokes equations. The resonant frequency can shift due to compressibility of the gas as it changes damping coefficient of an oscillation system [6].

### 3.2 Nonlinearity due to bias potential

To operate the resonators using the aforementioned circuit, an adjustable DC power supply is connected to the shuttle of a resonator and is used to establish a high motional current. However, non-linearity in resonating structures can result from high DC bias voltages, resulting in large oscillation amplitudes. The mechanical nonlinearity caused by the modification of effective spring constant of a 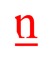 oscillation system [21]. The electrical nonlinearity usually occurs between plates or beams that experience large deflections. Accordingly, the electrostatic force is modeled as the expression of Taylor expansion [22]. The mechanically dominant nonlinearity results in a rightward bending of the resonant peak. In these cases, the amplitude versus frequency plots exhibit a hysteresis, which is also called the Duffing effect [23]. The Duffing effect is a nonlinear dynamic behavior that, in a single degree-of-freedom (DOF) system (such as a lateral resonator) can be modeled by:

(2) $$mx^{\prime \prime }+cx^{\prime }+k_{1}x+k_{3}x^{3}=F(t)$$

where

(3) $$k_{1}=m\omega_{r}^{2}$$

and

(4) $$c=m\frac{\omega_{r}}{Q}$$

Equation 2 is similar to Eq. (1), but differs by the stiffness coefficients. In this case, *k_1_* and *k_3_* are stiffness coefficients representing linear and cubic stiffness coefficients, respectively. The square coefficient is negligible due to the symmetric structure of the device. In Eqs. (2) and (3), *ω_r_* is the resonant frequency and *m* is the effective mass of the MEMS resonator that is mainly comprised of the proof mass. The drive force, *F*, is a function of the distance between interdigitated comb fingers of the proof mass and drive pad, that is,

(5) $$F(t)=\frac{1}{2}{\left(V_{P}+V_{i}\sin\omega t\right)}^{2}\frac{\partial C(x)}{\partial x}$$

By ignoring the infringing capacitance, the capacitance of electrode-to-proof mass per displacement is

(6) $$\frac{\partial C(x)}{\partial x}≅\frac{N\varepsilon_{0}h}{d}$$

**Figure 7.**
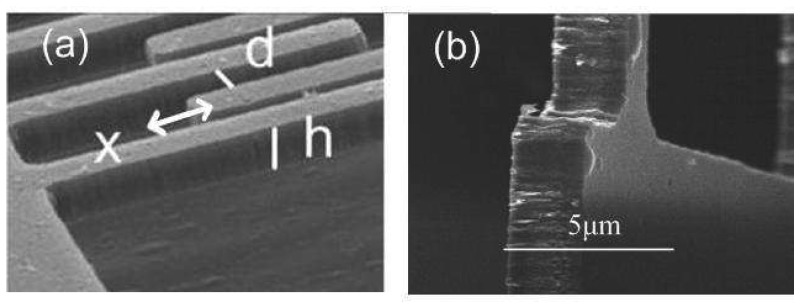
SEM micrograph of *Device D_f_1* used to determine (a) the dimensions of the interdigitated comb fingers: h is the thickness of SiC film; d is the gap between electrode and the interdigitated fingers; x is displacement and (b) the thickness of SiC film with highly oblique angle.

The dimensions of the interdigitated fingers are determined by SEM micrographs such at that shown in Fig. 7(a). In this micrograph, *h* is the thickness of the proof mass; *d* is the gap between electrode and the interdigitated fingers; *N* is the number of finger-gap; and *ε_o_* is permittivity in air. The cubic stiffness coefficient is difficult to quantify. An estimated value for the cubic stiffness coefficient can be obtained either by software simulation or by curve fitting of empirical results [24, 25]. Table 2 contains measured dimensions for *Device D_f_1*. From these dimensions and the equivalent model, the mass m is calculated to be 9.74×10^-11^ kg by estimating the beam thickness to be 2 μm using the SEM micrograph in Figure 7(b) and the SiC deposition process. From these data, the linear stiffness coefficient, *k_1_*, is determined to be 7.81 N/m and the damping coefficient *c* is about 4.5×10^-10^ N/(m·s^-1^) as by Eq. 3 and Eq. 4 as Q is 51,425 and ω_r_ is 1.77×10^-5^.

**Figure 8.**
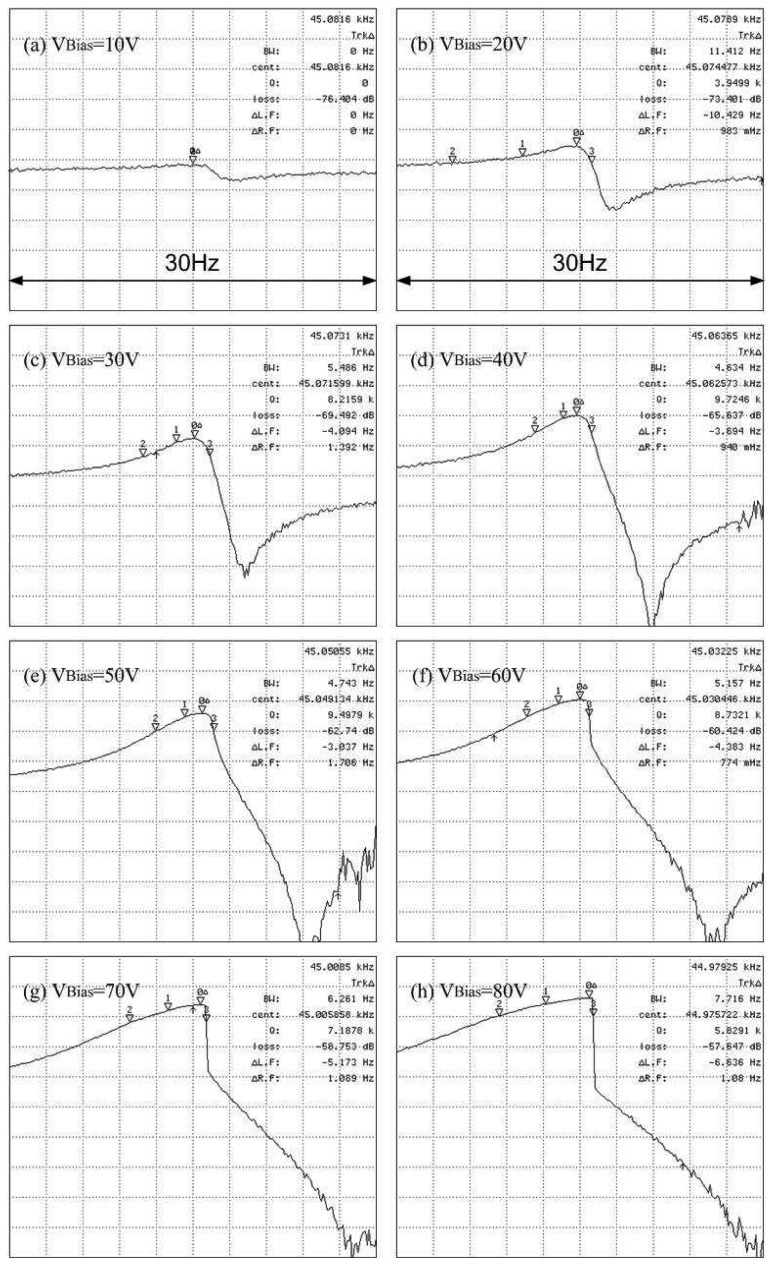
Magnitude plots for *Device D_f_1* for bias voltages ranging from 10 V to 80 V. The small inverse deltas mark the resonant frequency and frequencies at a 3 dB bandwidth. The vertical scale is 5 dB per grid.

Figures 8(a) to (h) are magnitude plots for the device under varied bias potential conditions. The resonator stiffness softens as the bias potential is increased. The Q of the resonator increases with bias potential due to amplification of its motional current. However, this value saturates as the resonator enters nonlinearity, namely, the Duffing effect, at around 40 V. The resonant frequency variation within this linear operation, i.e., bias potential ranging from 20 V to 40 V, is about -11ppm/V to -21ppm/V. By comparison, it has been reported that a 20 kHz, folded-beam polysilicon resonator exhibited a voltage coefficient variation of -54ppm/V [1].

**Figure 9.**
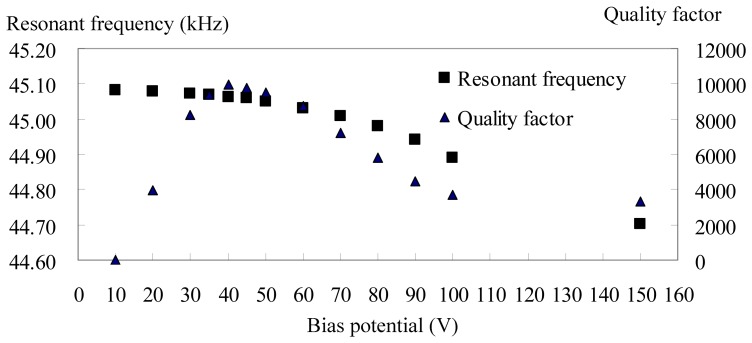
Nominal quality factor and resonant frequency as a function of bias potential for *Device D_f_1*.

Figure 9 is the nominal Q and resonant frequency as a function of bias potential for *Device D_f_1*. These values are read directly from the network analyzer. The drive voltage was fixed at 12.6mV and the ambient pressure was fixed at a pressure of 30 μTorr. The nominal Q in the frequency domain is related to the sharpness of the resonant peak and is given as the resonant frequency divided by the bandwidth of 3dB amplitude drop, that is,

(7) $$Q=\frac{f_{r}}{\Delta f_{3dB}}$$

The small inverse deltas of these plots mark the resonant frequency and the frequencies at a 3dB bandwidth. The vertical scale is 5dB per grid. The nominal Q of *Device D_f_1* increases with increasing bias potential in the low bias range because the amplified signal is so small that the 3dB bandwidth cannot be found. Because the bias is too high, the nominal Qs are distorted due to nonlinearity. The downward peak in the spectrum is caused by the equivalent parasitic capacitance associated with this electrical measurement technique. The parasitic capacitance mainly comes from the capacitance of the bonding wires.

### 3.3 Nonlinearity from drive voltage

**Figure 10.**
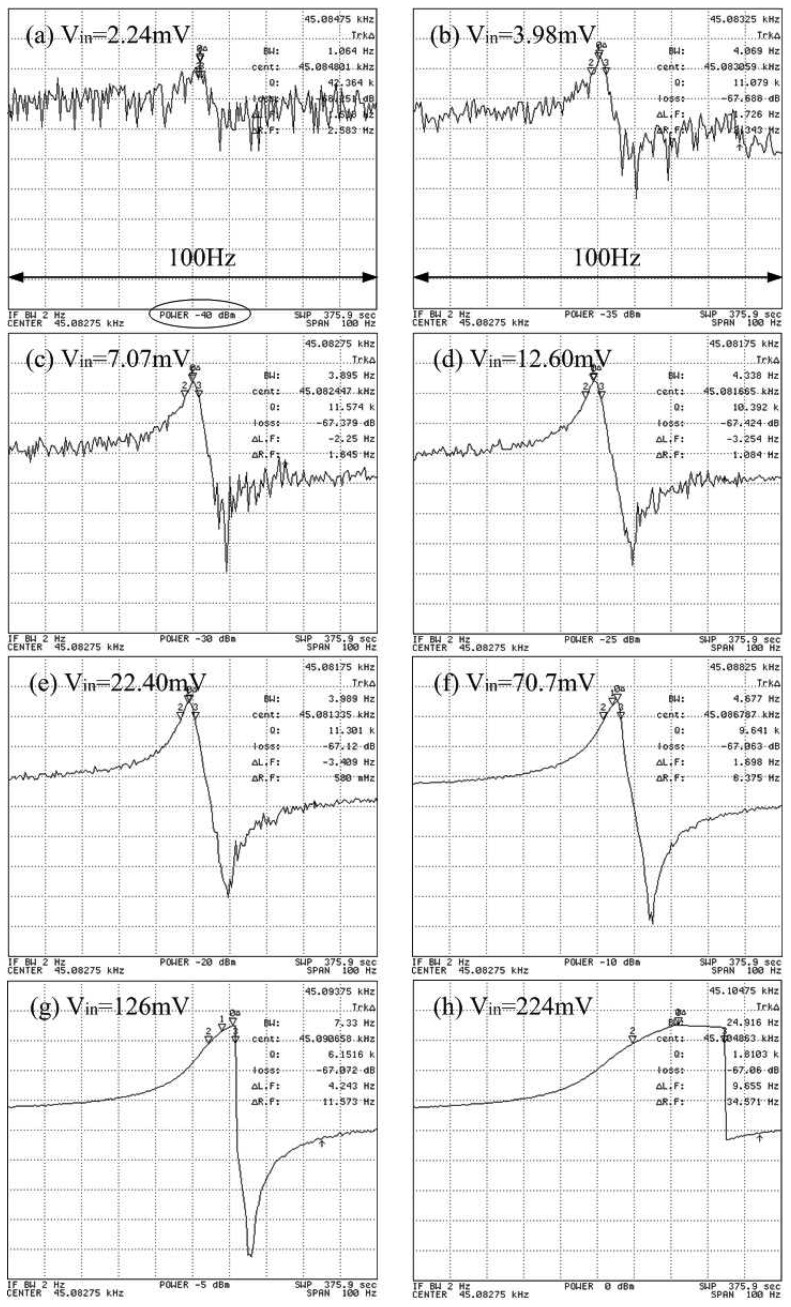
Magnitude plots for *Device D_f_1* for varied driving voltages ranging.

An AC drive voltage also causes nonlinearities in the performance of the SiC resonators. The oscillation amplitude of the resonators is directly determined by an applied AC voltage. If the AC voltage is too low, the noise level buries the small signal generated by the motional current. This phenomenon is seen from Figures 10(a) through (h), in which the DC bias is fixed at 40V. Figure 10(a) has the lowest drive voltage at 2.24 mV, but also has the highest jitter, i.e., noise, so that the signal is somewhat difficult to recognize. The network analyzer has two different scales for input voltage, one being power (dBm), shown under the plot and circled in (a), and the other being voltage (mV), as described on the top of each plot. Using nominal reduced Q as the measure to evaluate nonlinearity, the Q remains stable before AC is about 70.7 mV. The voltage coefficient due to drive voltage is about 1 to 4 ppm/V with a fixed DC bias of 40 V. However, this coefficient can vary with DC bias [21]. Although both high DC bias and AC drive voltage can cause nonlinearity, they do have different roles. The AC drive voltage supplies electrostatic force to the drive pad to attract the proof mass and DC bias is more like a current amplifier. Without bias, the AC drives force is excited only at the second harmonic from the expansion of Eq. 5. When bias voltage is nonzero, the expansion of Eq.5 contains the components with drive frequency of ω and twice of drive frequency 2ω. The force at the applied frequency is proportional to *V_p_*, since *V_p_* is typically much larger than *ν_i_*. Both bias potential *V_p_* and drive voltage *ν_i_* contribute to the electrostatic force and thus amplitude of the oscillation, which determines the mechanical nonlinearity.

### 3.4 Temperature effect

**Figure 11.**
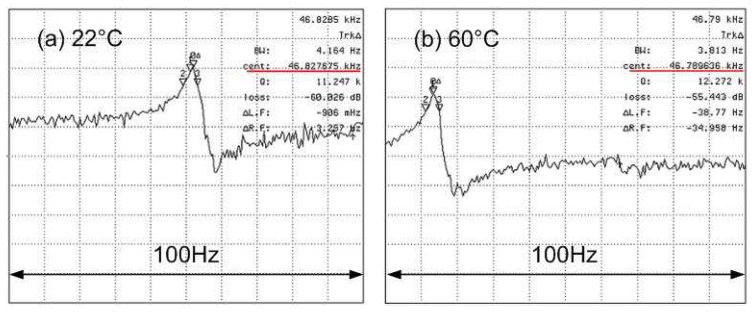
Magnitude plots at 22°C and 60°C for *Device D_f_5*.

**Figure 12.**
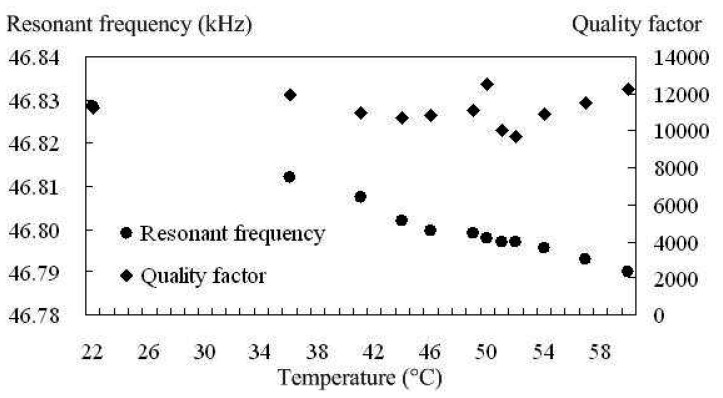
Plot of measured resonant frequency and Q versus temperature for *Device D_f_5*.

**Figure 13.**
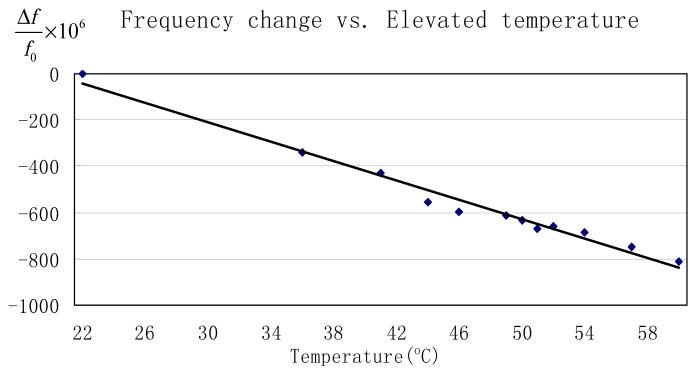
Normalized change in resonant frequency versus temperature for *Device D_f_5*. The temperature coefficient for the resonant frequency, as determined from these data, is -22 ppm/°C.

In a previous study where SEM was used to image the motion of SiC lateral resonators, it was observed that the resonant frequency decreased with increasing temperature due to tensile stress in SiC films and a decreasing Young's modulus with increasing temperature [19]. However, because SEM was used to determine resonator motion, the temperature dependence of the Q could not be determined. Figures 11(a) and (b) are magnitude plots at 22°C and 60°C, for *Device D_f_5* respectively. To avoid the nonlinear region associated with high amplitude oscillations, the bias voltage was fixed at 35 V and the drive voltage was held at 22.4 mV. Figure 12 plots the measured resonant frequency and Q versus temperature. The Q exhibits no significant change from 22°C to 60°C. However, the data show that the resonant frequency drops from 46,828 Hz to 46,790 Hz over the entire temperature range. From the normalized resonant frequency change versus elevated temperature plotted in Fig. 13, it is determined that temperature coefficient for the resonant frequency is -22 ppm/°C between 22°C and 60°C. This value may be slightly overstated because the actual temperature of the resonator may be higher than 60°C due to indirect contact temperature measurement method used in this experimental setup. By comparison, crystalline quartz has temperature coefficient that ranges from 14 to 100 ppm/°C depending on the crystalline orientation [26]. Our findings suggest that SiC exhibits a better temperature stability than quartz. However, a previous study found that the temperature coefficient of polysilicon resonators of similar design to be -10 ppm/°C [1], which is a factor of two better than the devices reported in this paper. This result does not imply that SiC as structural material is worse than Si, but rather that the substrate material may play a significant role in this case. The finite element analysis presented in Ref 19 showed that the resonant frequency change with increasing temperature relies on the interplay between the Young's modulus of the SiC resonator and induced stress in the supporting beams caused by the thermal expansion mismatch between resonator and substrate. The polysilicon devices characterized in Ref 1 were fabricated on Si substrates, thus eliminating thermal expansion mismatch between resonator and substrate. These findings suggests that while SiC may be a better material for extreme temperature operation, polysilicon may be the better selection for integrated microsystems based on Si IC's, where operating temperatures are maintained close to room temperature.

## 4. Conclusions

The results of this study show that SiC lateral resonators exhibit a resonant frequency shift along with ambient pressure due to a compact damping of gas-rarefaction effect much like similar devices made from polysilicon. The Q of kHz-frequency SiC lateral resonators increases with increasing DC bias potential but then decreases when the device is driven into nonlinearity as the resonator stiffness begins to soften as the bias potential is increased. The resonant frequency variation due to DC bias is about -11ppm/V to -21ppm/V, which is better than similar devices made from polysilicon. A low AC drive voltage leads to unacceptably high noise levels but its voltage coefficient is much lower than that for DC bias. The temperature coefficient of these resonators was determined to be -22 ppm/°C between 22°C and 60°C, which is comparable to quartz oscillators, but higher than similar devices made from polysilicon mostly due to the mismatch of the thermal coefficient of SiC structural material and underlying Si substrate.

## Figures and Tables

**Table 1.** Resonant frequency shift for *Device K1* under different pressures.

Pressure (Torr)	760	1	0.3	0.003	0.00003
Resonator frequency (Hz)	27977	28148	28155	28158	28162

**Table 2.** Dimensions of the *D_f_1* resonator

**Beam Width (m^2^)**	**Beam Length (m^2^)**	**Proof Mass Area (m^2^)**	**Truss Area (m^2^)**	**Beam Area (m^2^)**
2.10×10^-6^	1.00×10^-4^	2.40×10^-8^	6.90×10^-10^	1.60×10^-9^
